# Germline *RUNX1* variants in paediatric patients in a French specialised centre

**DOI:** 10.1002/jha2.594

**Published:** 2022-11-06

**Authors:** Cécile Liu, Paola Ballerini, Guillaume Nguyen, Zoia Mincheva, Bruno Copin, Boutheina Bouslama, Guy Leverger, Arnaud Petit, Rémi Favier, Hélène Lapillonne, Hélène Boutroux

**Affiliations:** ^1^ Sorbonne University, AP‐HP, Paediatric Haematology and Oncology Department Armand‐Trousseau Hospital Paris France; ^2^ Sorbonne University, AP‐HP, Laboratory of Haematology Armand‐Trousseau Hospital Paris France; ^3^ Sorbonne University, AP‐HP, French Reference Centre for Inherited Platelet Disorders Armand‐Trousseau Hospital Paris France; ^4^ Sorbonne University, AP‐HP, Service de Génétique et Embryologie Médicales Armand‐Trousseau Hospital Paris France

**Keywords:** genetic predisposition to disease, haematologic diseases, RUNX1

## Abstract

Familial platelet disorder with associated myeloid malignancy (FPD‐MM; OMIM 601399) is related to germline *RUNX1* mutation. The pathogenicity of *RUNX1* variants was initially linked to FPD‐MM phenotype, but the discovery of new variants through the expansion of genetic explorations in leukaemia is questioning this assertion. In this study, we add 10 families with germline *RUNX1* variant explored at Armand Trousseau Hospital for leukaemia diagnosis or thrombocytopenia, to the 259 described so far. Detailed description of their personal and family history of haematological pathologies allows identifying three phenotypes related to germline *RUNX1* variants: thrombocytopenia and/or malignant haematological disease with family history of haematological diseases, thrombocytopenia with no family history of haematological diseases and acute lymphoblastic leukaemia (ALL) with no family history of haematological diseases. In the latter phenotype, ALL characteristics involving *RUNX1* suggest the implication of germline variants in the onset of the malignancy.

## 1 INTRODUCTION

Acute leukaemia is the most common childhood cancer, representing 15%–30% of paediatrics cancers [1]. At present, few causes are related to leukaemia occurrence apart from rare predisposition syndromes [2]. Familial platelet disorder with associated myeloid malignancy (FPD‐MM; OMIM 601399) is related to germline *RUNX1* mutation. *RUNX1* encodes for the RUNX1 protein, which is a major transcription factor for haematopoiesis [3]. RUNX1 have three isoforms (RUNX1a, RUNX1b and RUNX1c). All isoforms have the runt homology domain, a critical region for its function by allowing binding to DNA [4], whereas only isoforms b and c have a complete C‐terminal. Complete deletion of *RUNX1* leads to fatal haematopoiesis failure in disease models [5]. FPD‐MM families, related to *RUNX1* mutations, regroup cases of thrombocytopenia and myeloid malignancies, with autosomal dominant transmission. About 35% of patients with FPD‐MM will develop haematological malignancies [6], including the recently reported B‐cell acute lymphoblastic leukaemia (ALL) [7, 8]. *RUNX1* variants are progressively being described, and pathogenic variants have been pooled in a recent review published by Brown et al., showing phenotype heterogeneity in FPD‐MM families [9]. The expansion of genetic explorations in leukaemia leads to the discovery of new germline *RUNX1* variants [10]. Diagnosing a constitutional variant predisposing to haematological malignancies may have an impact on the treatment choice but also on the relatives because of the hereditary component of FPD‐MM. Finding the connection between genotype and phenotype is crucial as it would help guide therapeutics, considering the possible risk of secondary cancers due to chemotherapy. Initially, germline *RUNX1* variants were classified as pathogenic if associated with FPD‐MM phenotype, but the discovery of new variants in cancer patients with no history of thrombocytopenia or familial haematological diseases is questioning this assertion. The pathogenicity of a variant can be inferred by using in silico risk prediction scores and databases like ClinVar [11] or VarSome [12]. Currently, ClinGen expert panel is considered a reference regarding *RUNX1* variants [13]. Describing new cases of germline *RUNX1* variants, including incidental ones, will help clinicians manage cases with likely predisposition mutations.

Here, we described 10 French families with germline *RUNX1* variants, explored at Armand Trousseau Hospital for leukaemia diagnosis or thrombocytopenia, with detailed description of personal and family history of haematological pathologies.

## 2 METHODS

Children with germline *RUNX1* variants followed in our centre for acute leukaemia or for thrombocytopenia were included retrospectively from 2016 to 2021. Informed consent was obtained from parents, and assent was obtained from patients, as appropriate, to participate in this observational study. Data were collected retrospectively from medical records in accordance with the ethical standards of our institution (number: 20220628143729). This study was conducted in accordance with the Declaration of Helsinki.

NGS testing was performed in all patients diagnosed with acute leukaemia and suspected constitutional thrombocytopenia.

Germline *RUNX1* variant was searched by NGS‐targeted capture analysis, in bone marrow for patients with leukaemia and in peripheral blood for patients with thrombocytopenia. For patients with leukaemia, germline origin was confirmed in bone marrow or peripheral blood samples collected after complete remission. To do this, a 1 μg of genomic DNA sample was fragmented with the bioruptor standard sonicator (Diagenode, Liège, Belgium) to an average size of 400 bp. Adapter‐ligation, amplification and indexation were then performed using the SeqCap EZ Library SR protocol (Roche, Basel, Switzerland). Sequence targets corresponding to 60 thrombocytopenia‐involved and thrombocytopenia‐candidate genes were captured and amplified in accordance with the manufacturer's recommendations. The enrichment probes were selected to target the coding exons of genes based on their RefSeq canonical ID. Enriched libraries were then subjected to 250 bases paired‐end sequencing (MiSeq; Illumina, San Diego, California, USA). Two different pipelines based on BWA [14] and Bowtie2 [15] alignment tools were used to map sequence reads back to the human reference genome (hg19), and SAMtools mpileup (0.1.17) [16], GATK UnifedGenotyper (1.3–21) [17] and Pindel (0.2.4) [18] were used to identify variants (SNPs/indels), which were annotated using the ANNOVAR program [19]. We further considered the variants identified in thrombocytopenic individuals if the allele frequency was below 0.008 (in Exome Variant Server controls and 1000 Genomes total and European controls), and if the depth was ≥50×. The analysed regions included coding sequences, UTR regions and 5 bp flanking the exons.

The mutations identified by targeted capture sequencing were checked by Sanger sequencing to confirm germline origin for patients with leukaemia or for FPD‐MM‐related patients.

Variants were named according to the Human Genome Variation Society (cDNA numbering uses +1 as the A of the ATG translation initiation codon, and protein numbering takes the initiation codon as codon 1), based on RUNX1c.

Germline *RUNX1* variants were classified using American College of Medical Genetics and Genomics (ACMG) and the Association for Molecular Pathology (AMP) criteria, adapted to *RUNX1* by ClinGen expert panel [13].

## 3 RESULTS

**FIGURE 1:**
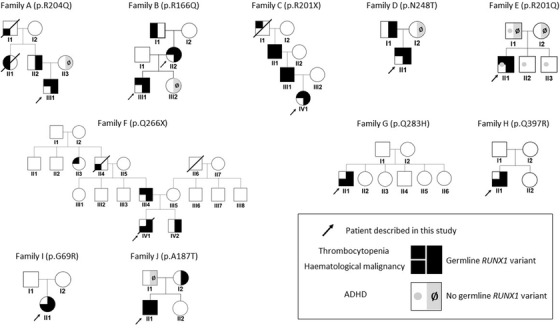
Description of the 10 pedigrees with germline RUNX1 variants

**FIGURE 2:**
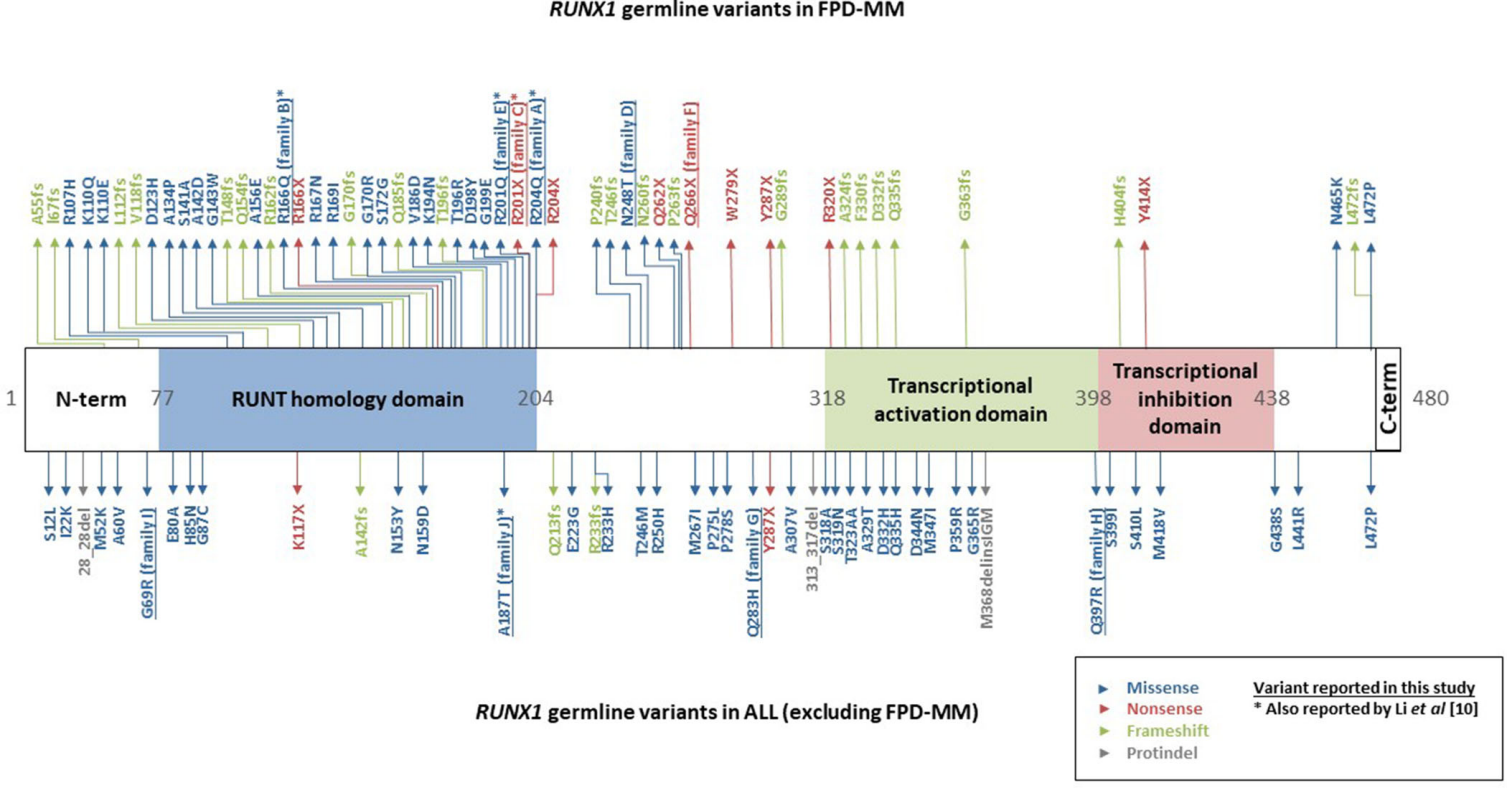
Localisation of the reported variants on RUNX1c based on previous publications [10]

Eleven patients, distributed in 10 families, met the inclusion criteria. Their pedigrees are detailed in Figure 1. *RUNX1* variants and the associated phenotypes are summarised in Table 1, and their localisations are indicated in Figure 2.

**TABLE 1:** Summary of the RUNX1 variants and associated clinical phenotypes in the 10 families

Family	Variant	Phenotype	Age of diagnosis (years)	Family history of haematological diseases	Pathogenicity according to ClinGen [13]	Pathogenicity according to ClinVar [11]	Pathogenicity according to VarSome [12]	ACMG/AMP criteria applied [13]	FPD‐MM
A	c.611G>A (p.R204Q)	Thrombocytopenia, urogenital malformations	13	Yes	Pathogenic	Pathogenic	Pathogenic	PS4 supporting (one proband meeting FPD‐MM phenotype), PM1 moderate (located in mutational hotspot), PM2 (absent from controls), PP3 supporting (computational evidence supports deleterious effect)	**Yes**
B	c.497G>A (p.R166Q)	Thrombocytopenia	II2 : 30 III1 : 3	Yes	Pathogenic	Pathogenic	Pathogenic	PS4 moderate (two probands meeting FPD‐MM phenotype), PM1 moderate (located in mutational hotspot), PM2 (absent from controls), PP3 supporting (computational evidence supports deleterious effect)	**Yes**
C	c.601C>T (p.R201X)	Thrombocytopenia	3	Yes	Pathogenic	Pathogenic	Pathogenic	PVS1 (null variant in a gene where loss‐of‐function is a known mechanism of disease), PS2 moderate (three proven de novo occurrences), PS4 strong (four probands meeting FPD‐MM phenotype), PM1 moderate (located in mutational hotspot), PM2 (absent from controls), PP3 supporting (computational evidence supports deleterious effect)	**Yes**
D	c.743A>C (p.N248T)	Thrombocytopenia, mental retardation, dysmorphic features	11	No	Not reviewed	Uncertain significance	Uncertain significance	PM2 (absent from controls)	**Uncertain**
E	c.602G>A (p.R201Q)	Thrombocytopenia, recurrent fever, ADHD	1	No	Pathogenic	Pathogenic	Pathogenic	PS2 supporting (one proven de novo occurrence), PS3 strong (well‐established functional studies supportive of a damaging effect), PS4 supporting (one proband meeting FPD‐MM phenotype), PM1 moderate (located in mutational hotspot), PM2 (absent from controls), PP3 supporting (computational evidence supports deleterious effect)	**Yes**
F	c.769C>T (p.Q266X)	AML, thrombocytopenia	16	Yes	Not reviewed	Not reviewed	Pathogenic	PVS1 (null variant in a gene where loss‐of‐function is a known mechanism of disease), PS4 moderate (three probands meeting FPD‐MM phenotype), PM2 (absent from controls), PP3 supporting (computational evidence supports deleterious effect)	**Yes**
G	c.768G>C (p.Q283H)	T‐cell ALL	14	No	Not reviewed	Uncertain significance	Uncertain significance	PM2 (absent from controls), PP3 supporting (computational evidence supports deleterious effect)	**No**
H	c.1190A>G (p.Q397R)	B‐cell ALL	14	No	Benign	Benign	Benign	PP3 supporting (computational evidence supports deleterious effect), BS1 strong (allele frequency is greater than expected for disorder), BS2 (observed in healthy adults)	**No**
I	c.205G>C (p.G69R)	B‐cell ALL	5	No	Benign	benign	Benign	BS1 strong (allele frequency is greater than expected for disorder), BS2 (observed in healthy adults), BP6 (classified as benign by ClinGen expert panel)	**No**
J	c.559A>G (p.A187T)	B/T‐cell ALL	5	No	Not reviewed	Uncertain significance	Likely pathogenic	PM1 supporting (located in well‐established functional domain RHD), PM2 (absent from controls), PP3 supporting (computational evidence supports deleterious effect)	**Uncertain**

### 3.1 Family A

Family A has a three‐generation history of haematological diseases. The index case (A:III‐1) presented with neonatal moderate thrombocytopenia (platelets count: 100 G/L), which subsequently fluctuated between 141 and 201 G/L. An unusual elevated thrombopoietin as well as a prolonged bleeding time was also present.

The medical history further included neonatal jaundice, urogenital malformations with hypospadias, cryptorchidism and bilateral kidney hypoplasia and cow's milk protein intolerance. The patient's paternal grandfather (A:I‐1) died of acute myeloid leukaemia (AML) secondary to myelodysplastic syndrome (MDS), and his paternal aunt (A:II‐1) had thrombocytopenia and died at age 34 from lymphoma. Both parents (A:II‐2 and A:II‐3) have a normal platelet count. Germline *RUNX1* variant c.611G>A (p.R204Q) was found in the index case (A:III‐1) and his asymptomatic father (A:II‐2).

### 3.2 Family B

The proband of family B (B:II‐2) was a young woman explored for a thrombocytopenia discovered during pregnancy at age 30. She had a platelet count varying between 60 and 150 G/L, easy bruising and mild postpartum haemorrhage, associated with increased thrombopoietin and prolonged bleeding time. Her father (B:I‐1) was also thrombocytopenic and recovered from AML, her daughter had a normal platelet count, whereas her son (B:III‐1) had a moderate thrombocytopenia varying between 100 and 130 G/L associated with delta granules impaired secretion and high levels of thrombopoietin. A slight hypereosinophilia between 0.6 and 1.3 G/L was also present in her son (B:III‐1). The NGS analysis revealed a germline *RUNX1* variant c.497G>A (p.R166Q) in the index case (B:II‐2) and her son (B:III‐1).

### 3.3 Family C

Family C has a four‐generations history of thrombocytopenia, and the index case (C:IV‐1), a 3 years old girl, was explored due to mild thrombocytopenia at 91 G/L with prolonged bleeding time.

The genetic analysis of the proband revealed a *RUNX1* variant c.601C>T (p.R201X), which was further diagnosed in her father (C:III‐1) and her paternal grandfather (C:II‐1).

Both of them had a history of MDS and thrombocytopenia (C:III‐1 and C:II‐1). The paternal great‐grandfather (C:I‐1) was also thrombocytopenic, but the genetic study could not be performed. Similar to B:III‐1, C:IV‐1 also presented mild hypereosinophilia between 0.5 and 1.4 G/L.

### 3.4 Family D

Patient D:II‐1 was incidentally diagnosed with thrombocytopenia at 72 G/L during the diagnostic work‐up of a moderate mental retardation combining facial dysmorphism and testicular ectopia. Subsequently, the platelet count dropped to 3G/L, and he developed epistaxis. Treatment with intravenous immunoglobulins was scarcely effective, but patient's platelets rose to the normal following thrombopoietin receptor agonist treatment by romiplostim.

Bone marrow smears inspection showed normocellular marrow, no megakaryocytic abnormalities or myelodysplasia signs but a significant increase of eosinophilic cells (7%), without peripheral blood eosinophilia. The platelet count remained normal 6 months after the last thrombopoietin receptor agonist injection, despite a prolonged bleeding time linked to the impaired delta granules secretion. There was no family history of haematological diseases. NGS analysis identified a *RUNX1* variant c.743A>C (p.N248T) in the proband (D:II‐1), which was also present in his asymptomatic father (D:I‐1).

### 3.5 Family E

Similarly, family E has no family history of haematological diseases or thrombocytopenia. The index case (E:II‐1) was born with pancytopenia, which resolved around the age of 4 months except for the thrombocytopenia. The platelet count was between 60 and 80 G/L, and he had frequent ecchymosis. From the age of 2–5 years, he also presented with idiopathic recurrent fever, which responded to a polyvalent immunoglobulin treatment. In addition, he has a mild cognitive disorder, also diagnosed in his two brothers and his father. Multiple bone marrow aspirations showed normocellular marrow, fluctuating dysmegakaryopoiesis with small and hyposegmented platelet precursors, associated with 5% medullar eosinophilia, but no peripheral blood eosinophilia. Germline *RUNX1* variant c.602G>A (p.R201Q) was found in the index case (E:II‐1) but not in his parents (E:I‐1 and E:I‐2). Thus, the attention‐deficit hyperactivity disorder (ADHD) in the index case (E:II‐1) and his father (E:I‐1) does not appear to be linked with the germline *RUNX1* variant, although the siblings have not yet been studied for the mutation.

### 3.6 Family F

The index case (F:IV‐1) was diagnosed with AML at age 16 because of general alteration and pancytopenia, without any known thrombocytopenia. The myelogram showed 45% malignant myeloblasts, dysgranulopoiesis but no dysmegakaryopoiesis. Bone marrow cytogenetic and molecular analysis showed 7q‐, trisomy 21, *RUNX1* and *TET2* mutations and overexpression of *MECOM*. Clinical interview unveiled a family history of haematological diseases with mild thrombocytopenia in his father (F:III‐4), myelofibrosis in his paternal grandfather (F:II‐4) and a possible thrombocytopenia in his paternal grandaunt (F:II‐3). NGS further confirmed the germline nature of *RUNX1* variant c.769C>T (p.Q266X) in the index case (F:IV‐1), his father (F:III‐4) and the asymptomatic brother (F:IV‐2). The patient (F:IV‐1) died of a second relapse of AML 2 months, and a half after 10/10 matched unrelated donor hematopoietic stem cell transplantation (HSCT) carried out because of early relapse after induction. Complications were central catheter‐related infections, persistent thrombocytopenia after induction and post‐transplantation lymphoproliferative disease (PTLD) linked to Epstein‐Barr virus.

### 3.7 Family G

Family G has no family history of haematological diseases. The index case (G:II‐1) was diagnosed with pre‐cortical T‐cell ALL at age 14, in the context of asthenia and cervical adenopathy, harbouring *NOTCH1, FBXW7* mutations, a partial deletion of *PTEN* and a *RUNX1* variant c.768G>C (p.Q283H), with an allelic frequency of 50%. The karyotype showed a deletion del(9)(p13‐24) and no known translocations. The germline nature of *RUNX1* mutation was confirmed in the complete remission bone marrow, which showed the persistence of 50% of c.768G>C mutated *RUNX1* alleles. Complete blood count has been normal since the end of chemotherapy 2 years ago, and no severe adverse event occurred during the treatment.

### 3.8 Family H

As in the G family, there was no family history of malignancy. Due to exertional dyspnoea, the index case (H:II‐1) was diagnosed with B‐other ALL. Molecular analysis revealed a *RUNX1* variant c.1190A>G (p.Q397R) with an allelic frequency of 50%. The patient also presented with eczema and mild asthma. The germline nature of the *RUNX1* mutation was further confirmed in a bone marrow sample obtained at the time of the disease complete remission. Complete blood count has been normal since the end of chemotherapy 1 year ago, and no severe adverse event occurred during the treatment.

### 3.9 Family I

The index case (I:II‐1) was treated for B‐cell ALL with lymphomatous presentation and *ETV6::RUNX1* translocation associated with partial deletion of a normal copy of *ETV6* and a supplemental copy of *RUNX1*. The ALL treatment has been complicated by extremely severe pancreatitis secondary to asparaginase in the context of germline *SPINK1* mutation, requiring treatment adjustment with consolidation with blinatumomab, discontinuation of asparaginase after induction and maintenance without corticosteroids due to diabetes mellitus. Germline *RUNX1* variant c.205G>C (p.G69R) was found on ALL NGS panel and confirmed after complete remission, with no family history of haematological anomalies. Complete blood count has been normal since the end of chemotherapy 1 year ago.

### 3.10 Family J

Family J has no family history of haematological diseases. The index case (J:II‐1) presented with isolated thrombocytopenia at 102 G/L discovered during asthma associated with eczema explorations. Biphenotypic B‐ and T‐cell ALL with *KMT2A* rearrangement was diagnosed 1 month later with increased thrombocytopenia at 25 G/L, cutaneous haemorrhagic symptoms, pancytopenia and hepatosplenomegaly. HSCT was indicated because of insufficient decrease of minimal residual disease and germline *RUNX1* variant c.559G>A (p.A187T), confirmed on fibroblasts. The same *RUNX1* variant was found in his asymptomatic mother (J:I‐2). Note that 5/6 unrelated cord blood transplantation was complicated by stage 1 acute cutaneous graft versus host disease treated with topical steroids and an alloimmune haemolytic anaemia at 3 months with a favourable outcome under corticosteroids and four injections of rituximab. Minimal residual disease is negative 6 months after HSCT.

## 4 DISCUSSION

In this study, we add 10 families to the 259 so far reported [20].

Three phenotypes related to germline *RUNX1* variant come out from these results: thrombocytopenia and/or malignant haematological disease with family history of haematological diseases, thrombocytopenia with no family history of haematological diseases, and ALL with no family history of haematological diseases.

Families A, B, C, E and F exhibit a phenotype of FPD‐MM with germline *RUNX1* variants residing in the runt domain. Apart from the variant found in family F, these germline variants were previously described in FPD‐MM [4, 9, 10, 21–23]. The pathogenicity of these variants can be affirmed because fitting with ClinGen, ClinVar and ACMG criteria.

A new *RUNX1* variant was identified in family D. The pathogenic role of the p.N248T *RUNX1* variant in the subject D:II‐1 can be questioned because of a strictly asymptomatic father carrying the same variant, a complete correction of the platelet count after treatment with thrombopoietin receptor agonists, no anterior similar case described and a protein mutation located outside of the runt domain, but persistent functional platelet abnormalities consistent with FPD‐MM. Immune thrombocytopenia (ITP) is usually a differential diagnosis of constitutional thrombocytopenia, and no cases of ITP have been described in the context of FPD‐MM.

Another relevant feature is marrow or peripheral blood eosinophilia in families B, C, D and E. Medullar eosinophilia was previously described by Kanagal‐Shamanna et al. [24], and the frequency of eczema in FPD‐MM [9] may suggest a role for *RUNX1* in eosinophilia and the atopy spectrum.

Index cases of families G, H, I and J had fortuitous discovery of germline *RUNX1* variant in the context of ALL diagnosis. In accordance with previous data [10], those variants were mainly located outside the runt domain with the exception of p.A187T.

Germline pA187T variant was found in a patient presenting with biphenotypic ALL with *KMT2A* rearrangement and a second acquired *RUNX1* mutation. This variant was first reported in a patient with B‐cell ALL by Li et al. [10], but no further functional testing was performed due to the lack of significant impact on transcriptional regulation. Additional somatic mutations are a known mechanism of malignant transformation in FPD‐MM, including second acquired RUNX1 anomalies [25, 26, 27]. Variant p.G69R is located in the N‐terminal region of RUNX1 and is considered benign, but it would be interesting to determine *ETV6::RUNX1* breakpoint to consider the possible influence of this variant in leukaemogenesis. These associations may suggest that *RUNX1* alterations are involved in the pathogenesis of B‐cell ALL.

The families G, H, I and J had no haematological history, but the platelet count was not obtained from all asymptomatic parents to exclude a possible undiagnosed thrombocytopenia.

Genetic counselling must take into account this incertitude concerning the link between ‘non‐pathogenic’ variants and ALL occurrence. Another question concerns a possible impact of *RUNX1* variants on acute leukaemia prognosis. Predisposition to haematological malignancies can lead to privilege HSCT, but four cases of treatment failures were described for now, with one deceased from PTLD [28], one graft failure [28] and two relapses after HSCT [28, 29]. The expanding number of reports on germline *RUNX1* variants in haematological malignancies will help to better evaluate patients’ prognosis and treatment strategies.

With the increase of multigene panels performed at ALL diagnosis, more fortuitous germline *RUNX1* variants will be discovered. Interpretation of these mutations is delicate, and the impact on the treatment could be crucial. Family history of haematological diseases including mild thrombocytopenia and functional platelet abnormalities must be tracked to diagnose FPD‐MM. Functional studies of RUNX1 could be interesting in case of a non‐described variant with no familial haematological antecedents. Detection of MYH10 expression in platelets [30] would be an interesting biomarker of lack of RUNX1 activity, suggesting the pathogenicity of *RUNX1* alteration. Additional analysis is underway to determine the possible pathogenicity of the variants described in this study, using MYH10 expression in platelets. The development of the RUNX1 database [20] will assist in the management of these new variants, by compiling and classifying germline *RUNX1* data.

Due to a 35% risk of developing haematological malignancies, close monitoring of FPD‐MM patients, with clinical examination and complete blood count, appears to be necessary. NGS surveillance in blood samples can be discussed because additional mutations are frequently associated with haematological malignancies with FPD‐MM [25, 26, 27]. The long‐term follow‐up of B‐ALL with non‐FPD‐MM *RUNX1* variant will help us decide between a novel phenotype and mere coincidence.

## AUTHOR CONTRIBUTIONS

CL wrote the manuscript, collected biological and clinical data and designed Figures 1 and 2. PB and GN wrote the manuscript, curated biological data and contributed to NGS data. ZM curated and contributed to NGS data. AP and RF contributed to NGS data and clinical patient information. BC contributed to NGS analysis. HB and GL supervised the study. All authors critically reviewed and approved the manuscript.

## CONFLICT OF INTEREST

The authors declare they have no conflicts of interest.

## FUNDING INFORMATION

This research received no specific grant from any funding agency in the public, commercial or not‐for‐profit sectors.

## Data Availability

The data that support the findings of this study are available from the corresponding author upon reasonable request.

## References

[1] Steliarova‐Foucher E , Colombet M , Ries LAG , Moreno F , Dolya A , Bray F , et al. International incidence of childhood cancer, 2001–10: a population‐based registry study. Lancet Oncol 2017;18:719–31. 28410997 10.1016/S1470-2045(17)30186-9 PMC5461370

[2] Kratz CP , Jongmans MC , Cavé H , Wimmer K , Behjati S , Guerrini‐Rousseau L , et al. Predisposition to cancer in children and adolescents. Lancet Child Adolesc Health 2021;5:142–54. 33484663 10.1016/S2352-4642(20)30275-3

[3] Yamagata T , Maki K , Mitani K . Runx1/AML1 in normal and abnormal hematopoiesis. Int J Hematol 2005;82:1–8. 16105753 10.1532/IJH97.05075

[4] Song W‐J , Sullivan MG , Legare RD , Hutchings S , Tan X , Kufrin D , et al. Haploinsufficiency of CBFA2 causes familial thrombocytopenia with propensity to develop acute myelogenous leukaemia. Nat Genet 1999;23:166–75. 10508512 10.1038/13793

[5] Okuda T , van Deursen J , Hiebert SW , Grosveld G , Downing JR . AML1, the target of multiple chromosomal translocations in human leukemia, is essential for normal fetal liver hematopoiesis. Cell 1996;84:321–30. 8565077 10.1016/s0092-8674(00)80986-1

[6] Sood R , Kamikubo Y , Liu P . Role of RUNX1 in hematological malignancies. Blood 2017;129:2070–82. 28179279 10.1182/blood-2016-10-687830 PMC5391618

[7] Mok MMH , Du L , Wang CQ , Tergaonkar V , Liu TeC , Yin Kham SK , et al. RUNX1 point mutations potentially identify a subset of early immature T‐cell acute lymphoblastic leukaemia that may originate from differentiated T‐cells. Gene 2014;545:111–6. 24792891 10.1016/j.gene.2014.04.074

[8] Six KA , Gerdemann U , Brown AL , Place AE , Cantor AB , Kutny MA , et al. B‐cell acute lymphoblastic leukemia in patients with germline *RUNX1* mutations. Blood Adv 2021;5:3199–202. 34424323 10.1182/bloodadvances.2021004653 PMC8405188

[9] Brown AL , Arts P , Carmichael CL , Babic M , Dobbins J , Chong C‐E , et al. RUNX1‐mutated families show phenotype heterogeneity and a somatic mutation profile unique to germline predisposed AML. Blood Adv 2020;4:1131–44. 32208489 10.1182/bloodadvances.2019000901 PMC7094007

[10] Li Y , Yang W , Devidas M , Winter SS , Kesserwan C , Yang W , et al. Germline RUNX1 variation and predisposition to childhood acute lymphoblastic leukemia. J Clin Invest 2021;131:e147898. 34166225 10.1172/JCI147898 PMC8409579

[11] Landrum MJ , Lee JM , Benson M , Brown GR , Chao C , Chitipiralla S , et al. ClinVar:improving access to variant interpretations and supporting evidence. Nucleic Acids Res 2018;46:D1062–7. 29165669 10.1093/nar/gkx1153 PMC5753237

[12] Kopanos C , Tsiolkas V , Kouris A , Chapple CE , Albarca Aguilera M , Meyer R , et al. VarSome: the human genomic variant search engine. Bioinformatics 2019;35:1978–80. 30376034 10.1093/bioinformatics/bty897 PMC6546127

[13] Luo X , Feurstein S , Mohan S , Porter CC , Jackson SA , Keel S , et al. ClinGen myeloid malignancy variant curation expert panel recommendations for germline RUNX1 variants. Blood Adv 2019;3:2962–79. 31648317 10.1182/bloodadvances.2019000644 PMC6849945

[14] Li H , Durbin R . Fast and accurate long‐read alignment with Burrows–Wheeler transform. Bioinformatics 2010;26:589–95. 20080505 10.1093/bioinformatics/btp698 PMC2828108

[15] Langdon WB . Performance of genetic programming optimised Bowtie2 on genome comparison and analytic testing (GCAT) benchmarks. BioData Min 2015;8:1. 25621011 10.1186/s13040-014-0034-0 PMC4304608

[16] Li H , Handsaker B , Wysoker A , Fennell T , Ruan J , Homer N , et al. The Sequence Alignment/Map format and SAMtools. Bioinforma Oxf Engl 2009;25:2078–9. 10.1093/bioinformatics/btp352 PMC2723002 19505943

[17] McKenna A , Hanna M , Banks E , Sivachenko A , Cibulskis K , Kernytsky A , et al. The genome analysis toolkit: a MapReduce framework for analyzing next‐generation DNA sequencing data. Genome Res 2010;20:1297–303. 20644199 10.1101/gr.107524.110 PMC2928508

[18] Ye K , Schulz MH , Long Q , Apweiler R , Ning Z . Pindel: a pattern growth approach to detect break points of large deletions and medium sized insertions from paired‐end short reads. Bioinforma Oxf Engl 2009;25:2865–71. 10.1093/bioinformatics/btp394 PMC2781750 19561018

[19] Wang K , Li M , Hakonarson H . ANNOVAR: functional annotation of genetic variants from high‐throughput sequencing data. Nucleic Acids Res 2010;38:e164. 20601685 10.1093/nar/gkq603 PMC2938201

[20] Homan CC , King‐Smith SL , Lawrence DM , Arts P , Feng J , Andrews J , et al. The RUNX1 Database (RUNX1db): establishment of an expert curated RUNX1 registry and genomics database as a public resource for familial platelet disorder with myeloid malignancy. Haematologica. 2021;11. 10.3324/haematol.2021.278762 PMC8561292 34233450

[21] Latger‐Cannard V , Philippe C , Bouquet A , Baccini V , Alessi M‐C , Ankri A , et al. Haematological spectrum and genotype‐phenotype correlations in nine unrelated families with RUNX1 mutations from the French network on inherited platelet disorders. Orphanet J Rare Dis 2016;11:49. 27112265 10.1186/s13023-016-0432-0 PMC4845427

[22] Tawana K , Wang J , Király PA , Zombori M , Csomor J , Al Seraihi A , et al. Recurrent somatic JAK‐STAT pathway variants within a RUNX1‐mutated pedigree. Eur J Hum Genet 2017;25:1020–4. 28513614 10.1038/ejhg.2017.80 PMC5567149

[23] Haslam K , Langabeer SE , Hayat A , Conneally E , Vandenberghe E . Targeted next‐generation sequencing of familial platelet disorder with predisposition to acute myeloid leukaemia. Br J Haematol 2016;175:161–3. 26525156 10.1111/bjh.13838

[24] Kanagal‐Shamanna R , Loghavi S , DiNardo CD , Medeiros LJ , Garcia‐Manero G , Jabbour E , et al. Bone marrow pathologic abnormalities in familial platelet disorder with propensity for myeloid malignancy and germline RUNX1 mutation. Haematologica 2017;102:1661–70. 28659335 10.3324/haematol.2017.167726 PMC5622850

[25] Antony‐Debré I , Duployez N , Bucci M , Micol J‐B , Renneville A , Boissel N , et al. Somatic mutations associated with leukemic progression of familial platelet disorder with predisposition to acute myeloid leukemia. Leukemia 2016;30:999–1002. 26316320 10.1038/leu.2015.236

[26] Yoshimi A , Toya T , Kawazu M , Ueno T , Tsukamoto A , Iizuka H , et al. Recurrent CDC25C mutations drive malignant transformation in FPD/AML. Nat Commun 2014;5:4770. 25159113 10.1038/ncomms5770

[27] Sakurai M , Kasahara H , Yoshida K , Yoshimi A , Kunimoto H , Watanabe N , et al. Genetic basis of myeloid transformation in familial platelet disorder/acute myeloid leukemia patients with haploinsufficient RUNX1 allele. Blood Cancer J 2016;6:e392. 26849013 10.1038/bcj.2015.81 PMC4771963

[28] Owen CJ , Toze CL , Koochin A , Forrest DL , Smith CA , Stevens JM , et al. Five new pedigrees with inherited RUNX1 mutations causing familial platelet disorder with propensity to myeloid malignancy. Blood 2008;112:4639–45. 18723428 10.1182/blood-2008-05-156745

[29] Buijs A , Poddighe P , van Wijk R , Van Solinge W , Borst E , Verdonck L , et al. A novel CBFA2 single‐nucleotide mutation in familial platelet disorder with propensity to develop myeloid malignancies. Blood 2001;98:2856–8. 11675361 10.1182/blood.v98.9.2856

[30] Antony‐Debré I , Bluteau D , Itzykson R , Morabito M , Droin N , Deswarte C , et al. MYH10 protein expression in platelets as a biomarker of RUNX1 and FLI1 alterations. Blood 2012;120:2719–22. 22677128 10.1182/blood-2012-04-422352

